# Acceptability of a microfinance-based empowerment intervention for transgender and cisgender women sex workers in Greater Kuala Lumpur, Malaysia

**DOI:** 10.7448/IAS.20.1.21723

**Published:** 2017-08-02

**Authors:** Priya Lall, Stacey A. Shaw, Rumana Saifi, Susan G. Sherman, Nuruljannah Nor Azmi, Veena Pillai, Nabila El-Bassel, Adeeba Kamarulzaman, Jeffrey A. Wickersham

**Affiliations:** ^a^ Faculty of Medicine, Department of Medicine, Centre of Excellence for Research in AIDS, University of Malaya, Kuala Lumpur, Malaysia; ^b^ Department of Social Work, Brigham Young University, Provo, USA; ^c^ Department of Health, Behavior and Society, Johns Hopkins University School of Public Health, Baltimore, MD, USA; ^d^ Social Intervention Group, School of Social Work, Columbia University, New York, NY, USA; ^e^ Yale University School of Medicine, Department of Internal Medicine, Section of Infectious Diseases, AIDS Program, New Haven, CT, USA

**Keywords:** HIV, transgender women, cisgender women, sex work, microfinance

## Abstract

**Introduction**: Cisgender and transgender woman sex workers (CWSWs and TWSWs, respectively) are key populations in Malaysia with higher HIV-prevalence than that of the general population. Given the impact economic instability can have on HIV transmission in these populations, novel HIV prevention interventions that reduce poverty may reduce HIV incidence and improve linkage and retention to care for those already living with HIV. We examine the feasibility of a microfinance-based HIV prevention intervention among CWSW and TWSWs in Greater Kuala Lumpur, Malaysia.

**Methods**: We conducted 35 in-depth interviews to examine the acceptability of a microfinance-based HIV prevention intervention, focusing on: (1) participants’ readiness to engage in other occupations and the types of jobs in which they were interested in; (2) their level of interest in the components of the potential intervention, including training on financial literacy and vocational education; and (3) possible barriers and facilitators to the successful completion of the intervention. Using grounded theory as a framework of analysis, transcripts were analysed through Nvivo 11.

**Results**: Participants were on average 41 years old, slightly less than half (48%) were married, and more than half (52%) identified as Muslim. Participants express high motivation to seek employment in other professions as they perceived sex work as not a “proper job” with opportunities for career growth but rather as a short-term option offering an unstable form of income. Participants wanted to develop their own small enterprise. Most participants expressed a high level of interest in microfinance intervention and training to enable them to enter a new profession. Possible barriers to intervention participation included time, stigma, and a lack of resources.

**Conclusion**: Findings indicate that a microfinance intervention is acceptable and desirable for CWSWs and TWSWs in urban Malaysian contexts as participants reported that they were ready to engage in alternative forms of income generation.

## Introduction

Since the first cases of HIV were detected in Malaysia in 1986, the epidemic has been largely driven by injection drug use with bridges to sexual transmission [1–4]. By 2014, 80% of all new HIV diagnoses in Malaysia were attributed to sexual transmission [2,5]. Cisgender women sex workers (CWSW) and transgender women sex workers (TWSW) are at increased vulnerability to HIV through sexual transmission via contact with clients. A recent respondent-driven sampling study found high rates of HIV among CWSWs (11.1%) and TWSWs (12.7%) in Greater Kuala Lumpur [6]. These groups have been marginalized through a process of stigmatization, discrimination, and criminalization [7–10]. As in most countries, sex work is criminalized in Malaysia [11], leaving CWSWs and TWSWs vulnerable to physical and sexual abuse from legal authorities. Structural conditions also leave TWSWs and CWSWs vulnerable to economic instability [12–14], with research highlighting that transgender women often experience difficulties entering stable forms of employment due to lack of qualifications and discrimination. Poverty inhibits sex workers’ use of condoms with clients [11,15–17]; evidence has shown that many sex workers acquiese to requests from clients to not use condoms in exchange for higher payments [18,19].

### Microfinance interventions to reduce HIV transmission

Microfinance describes a number of financial programmes aimed to alleviate poverty, including vocational training, micro-loans, and micro-savings programmes, to individuals who would otherwise not have access to these financial schemes through conventional channels (e.g. banks, loans) [20,21]. HIV prevention interventions with microfinance components [20–23] have demonstrated a reduction in HIV-related risk taking behaviours for beneficiaries [24] due to the prominent role that economic instability can play in HIV transmission [12–14]. For example, a large community-level randomized trial of such an intervention in South Africa reported significant reductions in condomless sex with casual sex partners [25,26].

Microfinance interventions targeting CWSWs have shown promise in empowering participants to develop additional sources of income [19,21–23] and reducing sexual risk behaviours; in one such intervention in Nairobi, two-thirds of participants reported having an operational small business by the end of the intervention [27]. In the Undarga programme, which combined vocational education with financial literacy and HIV prevention training for CWSWs in Ulaanbaatar (Mongolia) [22], beneficiaries reported having fewer paying sexual partners and were less likely to engage in unprotected sex with clients [21].

There are no known studies examining the impact of these types of interventions on TWSWs’ HIV risk profile; though evidence suggests that their behaviours are influenced by a similar set of factors to those of CWSWs [8,9,28]. Moreover, there are currently no reported microfinance interventions targeting either group in Malaysia. In order to address the complex HIV risk environment affecting sex workers’ behaviours in Malaysia, we evaluated acceptability of a proposed microfinance-based HIV-prevention intervention with additional training components on financial literacy (i.e. methods of budgeting) and vocational education. Qualitative data from in-depth interviews with CWSW and TWSW was used to examine acceptability of the proposed intervention on three fronts, including: (1) participants’ readiness to enter other occupations and the types of jobs they were interested in; (2) their level of interest in each component of the potential intervention, including training on financial literacy and vocational education; and, (3) possible barriers and facilitators to the successful completion of the intervention.

## Methods

### Setting

Acceptability of the proposed interventions were assessed through individual interviews with CWSWs (*n* = 19) and TWSWs (*n* = 16). Fieldwork was conducted in collaboration with three NGOs between March and November 2015. These NGOs provide HIV prevention and social services, such as shelter to marginalized populations, including street-based CWSWs and TWSWs. As these NGOs are directed by members of the transgender and sex work communities, they had strong networks within these groups and were knowledgeable of areas in which street-based sex workers usually procured clients. Participants were sampled from three districts within Greater Kuala Lumpur – including Chow Kit, Gombak, and Cheras – which were reported by NGO staff to have high concentrations of street-based and brothel-based sex workers.

### Sampling

We adopted an inductive research strategy for the sampling framework and topic guide, which were driven by the precepts of grounded theory [29]. Potential participants were initially approached by outreach workers from one of the three participating NGOs. These outreach workers used a convenient sampling approach as many participants worked on the streets on an ad hoc basis and, hence, were difficult to contact. To be eligible participants had to identify as either cisgender or transgender women and report using some form of drug in the last 12 months, including alcohol, heroin, morphine, cannabis or any amphetamine-type substance (ATS). An additional criteria for eligibility was that they had engaged in sex work in the previous 12 months and were fluent in either English, Bahasa Malaysia or Tamil. In accordance with grounded theory [29], the sample size was dependent on saturation of themes occurring during interviews.

### Data collection instruments

Prior to the in-depth interview, participants completed a brief, closed-ended questionnaire recording their demographic details, patterns in sex work (e.g. types of venues they worked at) and drug using behaviours. For the in-depth interviews, a topic guide was used to examine their previous occupations, experience in sex work, and interest in joining a microfinance intervention. Participants’ acceptance of a potential microfinance intervention were explored through questions gauging their readiness to engage in alternative forms of income generation, including the types of occupations they were interested in, and their interest in different components of the potential intervention. Over the course of fieldwork, interview guides were altered in accordance with themes emerging from the data. Interviews were conducted by members of the research team who were fluent in English and either Tamil or Bahasa Malaysia. Table 1 displays the structure of the interview guide.

**Table 1.** Structure of topic guide

Topic	Areas covered
Sex Work Experience	Drivers for entry into sex wok Experience of sex work Opportunities and costs associated with sex work
Patterns of drug use	Drug use history Drug use during sex work Impact of drug use on employment, income
Personal Finances	Personal financial management Long-term strategies to save money Barriers and facilitators to saving money
Microfinance Intervention	Interest in a microfinance-based intervention Types of occupation respondents were interested in Potential challenges respondents could face when in non-sex work occupations Potential challenges for completion of the intervention

### Ethics

This study received approval from the institutional review boards of University of Malaya (Malaysia) and Yale University (USA). Prior to study enrolment, participants were notified of the purpose of the study, their right to withdraw at any point, and that their confidentiality will be maintained. They were also informed of the risks of partaking in this study, which include discussing sensitive topics, such as drug use or sex work. In the event that participants expressed concerns regarding issues addressed during the interview, i.e. experience of interpersonal violence, they were referred to NGOs that could provide assistance. All participants provided informed consent. Confidentiality has been maintained through ensuring that identifying information is concealed, for instance participants were given false names. Interviewers received training on ethics and interview technique prior to and throughout the data collection process.

### Analysis

Interviews were conducted in Bahasa Malaysia (*n* = 20), Tamil (*n* = 8), and English (*n* = 7). Interviews conducted in Bahasa Malaysia or Tamil were first transcribed in the source language then translated by members of the research team. Data was checked by team members fluent in the source language for possible errors during transcription and translation.

Transcripts were analysed through Nvivo 11 [30], using grounded theory [29] as a framework of analysis; data was initially categorized through “open codes” developed by the leaders of the research team. These codes were further refined through a series of meetings with members of the research team. During these meetings core categories were identified and compared in order to generate themes [29]. Core categories were defined as codes repeatedly occurring from the data and are, thus, comparable across participants’ narratives. An example arising from this particular study would be when we discussed at length participants’ accounts of their drug use and further refined the coding schema to be reflective of its impact on their spending patterns. An audit trail was developed through team members’ own notes and memos over the course of analysis to ensure high inter-coder reliability [31].

## Results

Sample characteristics, stratified by gender identity, are reported in Table 2. Participants were on average 41 years old (SD = 12); and were mostly of Malay (42%), Indian (42%), or Chinese (9%) ethnicity. The majority of participants identified as Muslim (55%), followed by Hindu (27%), Buddhist (9%), Christian (6%), or Sikh (3%). Many participants reported not completing secondary school (45%) and less than half (46%) had children. Participants’ demographic traits differed according to their gender identity, with a substantially higher proportion of TWSWs reporting being single (87%), having no children (87%), and holding some form of secondary schooling qualification (60%) when compared to CWSW. Moreover, a larger percentage of CWSWs reported being widowed/divorced (72%) in comparison to their TWSW counterparts.

**Table 2.** Participants’ demographic characteristics according to gender identity

	Participants’ gender identity			
	Cisgender (*N* = 18)		Transgender (*N* = 15)	
Demographic characteristics	*N*	(%)	*N*	(%)
*Age (years)*				
24–29	1	(6)	5	(33)
30–39	8	(44)	4	(27)
40–49	2	(11)	3	(20)
50 or more	7	(40)	3	(20)
*Ethnicity*				
Malay	10	(56)	4	(27)
Chinese	1	(6)	2	(13)
Indian	7	(39)	7	(47)
Other	0	(0)	2	(13)
*Religion*				
Buddhist	1	(6)	2	(13)
Christian	2	(11)	0	(0)
Hindu	2	(11)	7	(47)
Muslim	13	(72)	5	(33)
Other	0	(0)	1	(7)
*Relationship status*				
Single	2	(11)	13	(87)
Has a partner	0	(0)	1	(7)
Married	3	(17)	0	(0)
Widowed	5	(28)	0	(0)
Divorced/separated	8	(44)	1	(7)
Number of children				
0	2	(11)	13	(87)
1–2	7	(39)	2	(13)
3 or more	9	(50)	0	(0)
*Level of education*				
Less than primary	3	(17)	1	(7)
Primary	1	(6)	0	(0)
Secondary (incomplete)	10	(56)	4	(27)
Secondary (complete)	4	(22)	7	(60)
Tertiary	0	(0)	1	(7)

Table 3 reports participants’ patterns of income generation, stratified by gender identity. Participants reported having engaged in sex work for an average of 20 years (SD = 12); although, both CWSWs (61%) and TWSWs (72%) reported receiving income from non-sex work forms of work, including cleaning services, entertainment, and night club promotion. Participants’ total mean monthly income was MYR 2235 (USD 521).

**Table 3.** Participants’ patterns of income generation according to their gender identity

	Participants’ gender identity			
	Cisgender (*N* = 18)		Transgender (*N* = 15)	
Patterns of income generation	*N*	(%)	*N*	(%)
*Age first started sex work (years)*				
16–19	2	(11)	9	(60)
20–24	4	(22)	4	(27)
25–29	4	(22)	2	(13)
30 or more	8	(44)		
*Amount of time engaging sex work (years)*				
Less than 1 year	2	(11)	0	(0)
1–9	0	(0)	7	(47)
10–19	9	(50)	2	(13)
20–29	3	(17)	4	(27)
30 or more	4	(22)	2	(13)
*Received income from other occupations in previous 6 months*				
Yes	11	(61)	13	(87)
No	7	(39)	2	(13)
*Involvement in sex work*				
Full time	9	(50)	3	(21)
Part time	6	(33)	10	(71)
Periodic	3	(17)	1	(7)
*Number of days engaging in sex work during a month*				
0–9	9	(50)	6	(43)
10–19	6	(33)	3	(21)
20 or more	3	(17)	5	(35)
*Income per month (MYR)*				
0–999	6	(33)	0	(0)
1000–1999	6	(33)	6	(43)
2000–2999	3	(17)	5	(36)
3000 or more	3	(17)	3	(21)

Table 4 shows rates of lifetime drug use, stratified by gender identity. The most commonly used drugs were amphetamine-type substances (ATS) (50%), primarily crystal methamphetamine, followed by cannabis (36%) and heroin (27%). Engagement in drug use differed according to respondents’ gender identity; with a higher percentage of CWSWs using ATS (61%) and/or heroine (44%) in comparison to TWSWs.

**Table 4.** Participants’ patterns of drug use according to their gender identity

	Participants’ gender identity			
	Cisgender (*N* = 18)		Transgender (*N* = 14)	
Patterns of drug use	*N*	(%)	*N*	(%)
*Types of substances used*				
Alcohol	11	(61)	11	(79)
Heroin	8	(44)	1	(7)
Morphine	2	(11)	2	(7)
Buprenorphine (Suboxone or Subutex)	3	(17)	0	(0)
Methadone	4	(22)	0	(0)
Benzodiazepines (dormicum, domi, diazepam, Xanax, Somes, Eramin 5)	3	(17)	4	(29)
Crystal Methamphetamine (ice, syabu, pil kuda)	11	(61)	5	(36)
Ecstasy (E, MDMA)	3	(17)	6	(43)
Ketamine (Special K)	0	(0)	1	(7)
Cannabis (Ganja, Boh, Balut, Marijuana)	6	(38)	6	(46)
Ketum (daun or air)	0	(0)	1	(7)

The in-depth interviews yielded three main themes regarding acceptability of a microfinance intervention: (a) participants were eager to engage in additional forms of income generation due to familial concerns and career aspirations; (b) interest in all proposed components of the intervention were driven by a desire to build their own business; and (c) potential challenges to developing businesses included a lack of financial resources, competition from other businesses, and fear of stigma.

### Readiness to engage in other forms of income generation

Both CWSW and TWSW expressed strong enthusiasm to participate in the proposed microfinance intervention, with many citing sex work as an unstable source of income. For many participants, their involvement in sex work has been intermittent, providing the opportunity to earn additional income when other sources failed to provide enough for them. Under these circumstances, sex work was framed as not being a “proper job” with opportunities for career growth but rather as a short-term and unstable form of income, often referred to as a source of “fast cash”. Puteri, a CWSW, aged 34, said “… they [sex workers] need to live a stable life. Maybe they feel they won’t work as sex workers forever”.

Participants described feeling locked out of employment opportunities in other professions, due mainly to the stigma of sex work and drug use. TWSW participants’ employment opportunities were further constrained by proscriptive gender roles in the Malaysian context. For example, many TWSW reported being able to find work in positions typically occupied by females, including bridal make-up artist, or cosmetics sales. Unfortunately, these positions were generally low-wage and vulnerable to seasonal shifts in market demand. For TWSW in particular, access to stable employment opportunities were limited due to implicit and explicit discrimination against transgender persons, making sex work one of the few reliable sources of regular income for them.

I would like a government job but it is hard for transgender people like me… I am interested [in finding other work]. But in job interviews with transgender people like us, many employers reject our applications.

Nor Naba, age unknown, TWSW

Employers discriminate against people with different gender identities like transgender and transsexuals but it seems as if they are not discriminating against [cisgender] females. Most of the time in Malaysia employers are only discriminating against transgender and transsexual people.

Kamu, 50 years old, TWSW

Drug use was also reported as a major obstacle to stable employment and income generation by both CWSW and TWSW. Participants reported being unable to work due to symptoms that they associated with addiction, including fatigue, nausea and body aches. Selly, a 58-year-old CWSW, who reported using heroin on a regular basis, stated, “Now I have back pain. when I’m high I feel stronger. When I’m not high, I can’t even sit up. [I]can’t even lift a feather.”

The impact of drug use on securing and maintaining employment was further compounded by the stigma associated with being a person who uses drugs. When applying for traditional employment opportunities, they report being stereotyped as untrustworthy and as criminals, instead of as hard-working people seeking employment. Nor Amal, a 34-year-old CWSW, stated, “Not all drug users are bad, not all drug users love to steal. But in the eyes of the public, ‘the normal people,’ when they find out that someone is a drug user like me…they will be like, ‘mind your shoes, drug users are walking by…’”. Beyond the stigma of drug use, many participants also reported employers’ ability to conduct criminal background checks as a major barrier to employment. For example, participants who had been previously arrested and convicted for drug-related crimes were faced repeated rejection from employers who discovered their arrest and conviction during checks of their criminal history, which are permitted under Malaysian law. One participant reported having been terminated by her employer after they learned of her previous arrest for drug use.

Participants’ motivation for engaging in alternative forms of income generation varied by gender identity. Among CWSWs, interest in microfinance and other forms of employment was underpinned by familial concerns and the stigma of sex work. Regardless of age, participants who stressed familial concerns reported that they entered sex work as a means to secure an income. Under these circumstances, microfinance is an opportunity to generate a reliable income, which could yield greater financial stability not only for them, but also their dependants, such as children and other relatives:

[For] My future… I can target [to start a small business]… from starting with a stall, I will have my own targets. I will target to open a restaurant. So it’s like for my own future. If it’s not for my future, it’s for my child’s future. If I can expand my business, then I can leave it to my child. At least my child will not have to go through hardship when she’s all grown up. Who knows she might go to the university, at least my business can back the plan up.

Nor Amal, 34 years old, CWSW

My dream, is as how people would say, to change. I want to open my own business. Let anyone say anything, never mind it’s okay. Even if it is a small business it’s okay. As long as I can survive until I die, for my children, that is enough. We are getting old each day. Children are also growing up fast.

Puteri, 34 years old, CWSW

Some CWSWs were motivated to partake in the microfinance intervention as a means to escape the stigma associated with engagement in sex work. One such participant perceived participation in the potential microfinance intervention as an opportunity for other sex workers to develop “a new life… A different life, not selling themselves, to become someone different”. They frequently described their participation in sex work as immoral, illustrating how shame and stigma can become internalized. Some participants argued that their engagement in sex work was reflective of their overall character. Another participant described a similar association between sex work and her own morality:

I do not want to prolong this sex work, which I am doing, I hope to stop [working as a sex worker] till here… Until now, if I have a chance, of course I would want the best. I do not want to close my eyes and be dead on the road. I do not want that. For example like the saying goes, who is not scared of death? Eventually everyone will die. It is not that I am scared to face death, I am ashamed to face God because I am living an immoral life. That’s the sad part.

Mysha, 57 years old, CWSW

For TWSW, however, long-held career aspirations were a major driver of interest in new forms of income generation and microfinance. Indeed, some had ambitions to employ others or provide a livelihood for family members. For example:

If I have the capital I will surely open a business. Even now I am looking at what business to open… That is my aim, make a lot of money and open your own business…. It’s in my mind always. [I] want to do this and that, but [I do not have the] budget yet. Have to work hard, have to do all work, to find the budget.

Aisha, 30 years old, TWSW

I like dance so I want to achieve something [from dancing]… Many transgender [do] not achieve [anything]. They do not have any targets to achieve something. They want to make money, make money, make money. For what they make money? After they earn the money they never put it inside [a bank]. So achieve. If Judie achieve in Indian classical dance, after I’m dead they say, ‘Judie’s a classical dancer, you know. She’s dead already. She’s a good dancer’. You know people talk about me. After five years, I have a dance studio, they come, the teacher say, ‘This is a professional dancer, Judie transgender, she dead in this year, born this year. She’s a transgender. They respect me.’ That I want. I want to achieve something in my profession.

Judie, 26 years old, TWSW

Finally, a small number of TWSWs noted age as a possible motive for participation; acknowledging that older sex workers seemed to generate less income than their younger counterparts. Stella’s motivation as a 57-year-old TWSW stemmed from the fact that she was not earning enough to cover her living expenses and she had few skills valuable in other areas of the labour market. She stated, ‘Now I’m old it’s so difficult to get money… One night I could get MYR700-plus, now I’m too old to earn that’. Meanwhile, younger TWSWs were wary of engaging in sex work till an old age as they witness older sex workers experiencing poverty. Hence, they perceived economic empowerment activities as an opportunity to develop skills necessary to acquire a small business through the capital they generated through sex work.

### Participants’ interest in different components of the potential intervention

Two participants stated that they would not be interested in participating in a microfinance intervention as they believed that they were not ready to invest time to focus on learning new skills. Jane, a 49-year-old TWSW, said that she was not interested “because when we have invested in these learning skills [from the microfinance course] for a long time, we have to focus on it completely. But I just can’t right?”. Others were eager to participate in a microfinance intervention with the hope that it would enable them to initiate a small business. Their interest in different components of the intervention, including training in professional skills and financial management, varied. Most participants expressed a high level of interest in the intervention and the training offered (*n* = 17) and were able to identify initial steps needed to enter a new profession. The remaining participants were either interested in all components of the intervention but were not sure how to enter a new career (*n* = 7), or were interested in one intervention component, primarily skill building (*n* = 9). Interviewees in the latter category were younger, hence, were still able to earn a substantial amount from sex work. Their interest in this component of the microfinance intervention was motivated by their long term goal to save enough money to initiate a business.

The vocational training component of the microfinance intervention was the aspect of greatest interest, in anticipation that it would lead to stable income generation. Both CWSWs and TWSWs sought to initiate a small enterprise within industries of catering, cosmetology, tailoring or entertainment. For participants who were interested in developing an enterprise in these three industries, the primary business option envisioned was to open a food stall or a beauty salon. A few participants were already working part-time selling food in local markets. For those who were interested in developing a career in entertainment, usually as a dancer, their primary business option was to set up a studio of their own. Participants noted other potential industries which sex workers would be interested in, including airline services, insurance, security, or health fields; work in a factory, hotel, or supermarket; or work in an office or non-governmental organization.

Experience with non-sex work employment and financial management varied greatly, pointing to the need for individualized supports. Some participants reported their financial management skills were sufficient but others talked about the importance of saving and business development, seeking access to financial management skills. A few such participants argued that they were unable to sufficiently cover their living expenses as they lacked the financial skills to budget and save:

Maybe they save money on that because we do sex work, we don’t know how to keep the money. We buy things whether it is worth it or not. We must think twice three times and then they buy. The changes must happen to them then they know how to. You see for me also every time I go to the supermarket I go and look for food only.

Aliya, 48 years old, CWSW

Yes, some sex workers will be interested. I know this because I have a friend who earns MYR7,000 but has no savings at all.

Dana, 45 years old, TWSW

If you don’t control it (spending of money), you follow your own desires, with no strategies, no plans, and you do things as you please, even if you earn MYR10,000 a month, you will not find it suffice. So from there, I will budget for a car.

Nor Amal, 34 years old, CWSW

### Potential barriers and facilitators to participating in microfinance intervention

Participants reported limited time to attend classes as many engaged in sex work on a part-time basis to supplement income from their primary jobs. They often worked in their primary job during weekdays and in sex work when needed during the evening, leaving a small window of time to attend training workshops. Aisha, a 30-year-old TWSW, reported that she was available only for a few hours on the weekend as on “Saturday, Sunday, there is definitely no leave because there are a lot of customers on these days”. Consequently, concern was expressed over being unable to meet basic financial needs if training occupied time usually allotted for sex work.

Other barriers included responsibilities for care of children and lack of access to public transportation. Unlike financial and time constraints, these barriers were perceived as manageable, especially if interviewees were keen on attending workshops. Nor Amal, a 34-year-old CWSW, noted, ‘I will want [to participate] really, because that’s what I’m interested in. If I’m not interested in it, then I will give excuses. I will give a thousand reasons’.

Possible barriers to success in a new, non-sex work occupation were described in terms of lack of resources and knowledge of how to ensure sustainability of small businesses. Participants often commented that although they had developed a business plan, they were unable to establish it as they did not have the capital. These interviewees reported:

I want to rent a space at a Ramadhan bazaar. I planned to make some Kuih [sweets]… I cook myself and then open a stall. But when I asked [about a stall], I learnt that at least you will need to have MYR1000 and that does not include license. If you apply for a license and include the cost of license; it will be over MYR1000, some reach over MYR2000, but there are (some licenses for) also less than MYR1000. I don’t know. I’m also confused which license is right or not.

Nor Amal, 34 years old, CWSW

I want to learn how to save- like [how to] collect money and make a business. I don’t want to just die like this (as a sex worker). I want to save money to open a business. At least when I have all of the money set aside, I want to open a business.

Aadila, age unknown, TWSW

Competition from other businesses were also perceived as a challenge to establishing and sustaining a small business. Some participants were not interested in partnering with others in the community in business ventures; due, in part, to a lack of trust among networks of sex workers. Dana, a TWSW, aged 45-years-old, stated, “I know some families can’t help [with a business] but mine can and we will help each other at all times. Not like friends [other sex workers], they will cheat you eventually.”

Participants were also concerned about stigma, which influenced perspectives on training as well as future business opportunities. This sentiment points to the negative influence judgment and stigma towards sex workers could have within a training framework, suggesting a degree of trust and familiarity may be gained when supports are provided by other sex workers. Concerns about discrimination when seeking to operate a business may severely limit actual and perceived economic opportunities outside of sex work. Being HIV positive was described as an added layer of stigma that may drive customers away, as Hazima, a 59-year-old TWSW, said, “If people know I have HIV they won’t come to my restaurant”. Those experiencing additional stigmatized identities may need added layers of support as they seek to develop effective business strategies.

Participants argued that they had already developed strategies for coping with stigma as they faced regular prejudice and discrimination in their daily lives. Coping strategies were part of a cognitive process in which they sought to “ignore” stigmatizing behaviours from others in their community. They believed that they were able to develop these cognitive processes from a position of strength fostered from their continual determination to leave sex work.

Stigma is a challenge; we need to be patient… that is our patience. If our patience is challenged, we would not be there. Let anyone say what they want to say. When they are tired, they will keep quiet. I will turn a deaf ear.

Mysha, 57 years old, CWSW

Oh, this [discrimination] is all normal. It is not a big thing for me at all, I don’t make a big fuss. I don’t feel anything… Because we can’t be noticing all of this. It happens when you are young and old. Sometimes, people always like to discriminate. We should just ignore them.

Jane, 45 years old, TWSW

We [other sex workers] need to have a strong spirit, too, don’t get upset over what people have to say. The most important is, straight to the point, we, what we are, what is our aim, we just carry on. No looking left or right, just say what we need to speak. This is a challenge we get from society.

June, 33 years old, TWSW

Another cognitive process that participants adopted was to draw inspiration from role models in their community who had left sex work for another profession. These role models illustrated that it was possible to overcome barriers associated with stigma to develop a sustainable business. A few role models would offer them advice on how to initiate a business. They reported:

Yeah, she [a former sex worker] is my friend for life. She has supported me for so long as a good friend. She cannot stand to see me work like this [as a sex worker]… She is the one who taught me how to fry the bananas, fish crackers and so on.

Puteri, 34 years old, CWSW

For me, I am not scared of discrimination because I see now, a lot of transwomen who run successful businesses. For example, Fatima.

Aisha, 30 years old, TWSW

Other participants were planning to take more direct actions to address barriers related to stigma; a few CWSWs reported that they would initiate their micro-enterprise in a location where their previous history of sex work is unknown. TWSW participants argued, on the other hand, that such barriers could only be addressed through interventions targeting stigma against sex work.

## Discussion

This study represents the first in-depth evaluation of the feasibility and acceptability of a microfinance-based HIV-prevention intervention for cisgender and transgender women sex workers who use drugs in Greater Kuala Lumpur. Participants’ socio-demographic characteristics differed slightly from that of other studies charting behaviours of sex workers in other South-East Asian countries, in which participants were often younger [32–35]. Moreover, the socio-demographic composition of the sample differed from that of the general Malaysian population in terms of age and ethnicity. According to current population estimates generated by the Department of Statistics, the median age of Malaysian citizens is 28 years old and less than a quarter of the population were of Indian ethnicity [36]. Participants socio-demographic characteristics were, nonetheless, similar to those displayed in a respondent-driven sampling study of CWSWs and TWSWs conducted in Greater Kuala Lumpur [6], in which the average age of participants was 37 years old and close to a quarter of the sample (22%) were of Indian ethnicity.

Overall, participants expressed a high level of readiness to engage with a microfinance programme. Both CWSW and TWSW were interested in developing their own small enterprises, mostly in the sectors of retail, catering, cosmetology, and entertainment. As many interviewees already had experience managing their own business, they were cognisant of steps needed to initiate their own enterprise but did not know how to make it sustainable. These results indicate that rather than offering beneficiaries’ vocational training for specific occupations, individualized support for development of sustainable businesses should be offered. Microfinance interventions in other countries offering similar types of business-related services to CWSWs have been proven to reduce beneficiaries’ risk behaviours through offering financial alternatives. The Pi Project [23] in Chennai (India), which offered street-based CWSWs training on tailoring of canvas bags, produced promising results with participants reporting significant increases in income and decreasing numbers of clients 6 months after the intervention.

Participants were, nevertheless, aware of possible barriers to completion of the microfinance intervention. The most prescient barriers were time-related as many participants worked part-time in other occupations, for example as a cleaner, which left a window of a few hours a week to attend classes. Moreover, many participants lacked capital to initiate a small business. These particular barriers could be ameliorated through reimbursing potential beneficiaries for time spent in training sessions [23] and offering micro-credits upon completion of the course [18,22].

Potential barriers to implementation of a microfinance intervention differed according to gender identity; the most pertinent for TWSWs being transphobia. Participants’ accounts pointed to a few possible methods for ameliorating stigma including strengthening of cognitive processes developed for coping with everyday discrimination and drawing inspiration from others in their community who established careers outside of sex work. These coping strategies could be incorporated in the microfinance intervention as a psychosocial component in the form of peer mentoring from prominent sex work activists and entrepreneurs.

A potential challenge for CWSWs could be addiction to ATS or heroin. This issue is pertinent among CWSWs in South-East Asian countries as recent research has highlighted that consumption of ATS is prevalent in the CWSW population in Cambodia [37,38], Vietnam [39] and Myanmar [40]. One such study conducted in Vietnam highlighted that CWSWs held misconceptions on ATS, believing that it was less addictive than heroin. Participants who reported symptoms of addiction seemed to exhibit high levels of interest in the microfinance intervention. Despite this intention, these participants may experience difficulties completing the intervention due to ongoing drug use; thus, underscoring the importance of linking future prevention strategies to local service, including medication assisted treatment through methadone or other opiate agonist therapy. (See Appendix 1 for a table charting different components of the potential intervention.)

### Limitations

The research for this paper had a few limitations; namely, that a convenience sampling approach was adopted owing to difficulties in approaching street-based sex workers who may engage in sex work on a sporadic basis. As outreach workers from collaborating NGOs sampled participants, it was also not possible to collect data on those who declined to be interviewed. Additionally, interviews conducted in languages other than English were not translated according to Brislin’s model, which is deemed to be the most accurate method for translating textual data. Possible errors in translation and transcription were mitigated through regular checks conducted by team members fluent in both languages. Finally, it is important to note that owing to the qualitative nature of this particular study, it is unlikely that the findings would be generalizable to the rest of the sex worker population in Malaysia [41,42]. As noted in the methods, participants were selected by means of theoretical sampling, i.e. their ability to provide information that would be pertinent to the development of a potential intervention [43].

## Conclusions

Findings of this study indicated that a microfinance intervention would be acceptable to CWSWs and TWSWs who engage in drug use in the urban Malaysian context as participants reported that they were ready to engage in alternative forms of income generation and expressed a strong interest in partaking in the potential intervention. The microfinance component of this particular intervention posed to meet the needs of TWSWs as well as CWSWs; both of whom noted a gap in knowledge in developing a sustainable small business.

Additional components may need to be added to the intervention to accommodate for the unique needs of TWSWs and CWSWs. These groups experienced stigma differently, many CWSWs had internalized stigma related with sex work, which was apparent by the fact they described their occupation as deeply shameful. They were, thus, unwilling to openly associate with other sex workers, which would sometimes curtail their ability to access services. This particular challenge further highlights the need for an additional psychosocial component to bolster potential beneficiaries’ ability to engage in non-sex work related income-generating activities.

Meanwhile, TWSWs perceived transphobia as a potential barrier to initiating their own enterprises. Hence, the incorporation of structural components, in the form of stigma reduction activities in the community, could potentially improve implementation of the microfinance intervention. Structural interventions have shown promise in addressing environmental factors influencing risk behaviours [44]. For example, the Sonagachi Project in Kolkatta, India, trained CWSWs to educate their peers on condoms and collectivized members of their community to mobilize against harassment and other forms of violence [45–47]. Data from longitudinal studies have indicated that beneficiaries’ levels of HIV knowledge, autonomy, financial security, and rates of condom use have significantly increased when compared to their non-participant counterparts [45].

## Notes

To access the supplementary material to this article please see Supplementary Files under Article Tools online.

## References

[1] Bazazi AR, Crawford F, Zelenev A, Heimer R, Kamarulzaman A, Altice FL. HIV prevalence among people who inject drugs in Greater Kuala Lumpur recruited using respondent-driven sampling. AIDS Behav. 2015;19(12):2347–10. 26358544 10.1007/s10461-015-1191-y PMC4743744

[2] UNAIDS, *The Global AIDS Response Progress Report* 2014: Malaysia., in *Country Progress Report*. 2014, UNAIDS: Malaysia.

[3] Mathers BM, Degenhardt L, Phillips B, Wiessing L, Hickman M, Strathdee SA, et al Global epidemiology of injecting drug use and HIV among people who inject drugs: a systematic review. Lancet. 2008;372(9651):1733–45. 18817968 10.1016/S0140-6736(08)61311-2

[4] Dokubo EK, Kim AA, Le L-V, Nadol PJ, Prybylski D, Wolfe MI HIV incidence in Asia: a review of available data and assessment of the epidemic. AIDS Rev. 2013;15(2):67–76. 23681434

[5] Health, M.M.o National strategic plan for ending AIDS: 2016-2030. 2015. Putrajaya, Malaysia: Malaysia Ministry of Health; 2016.

[6] Wickersham JA, Gibson BA, Bazazi AR, Pillai V, Pedersen CJ, Meyer JP., et al Prevalence of Human Immunodeficiency Virus and Sexually Transmitted Infections Among Cisgender and Transgender Women Sex Workers in Greater Kuala Lumpur, Malaysia: Results From a Respondent-Driven Sampling Study Sex Transm Dis. 2017;doi:10.1097/olq.0000000000000662. PMC5636657 28708696

[7] Amaro H, Raj A, Reed E Women’s sexual health: the need for feminist analyses in public health in the decade of behavior. Psychol Women Q. 2001;25(4):324–34.

[8] Gibson BA, Brown S-E, Rutledge R, Wickersham JA, Kamarulzaman A, Altice FL Gender identity, healthcare access, and risk reduction among Malaysia’s mak nyah community. Glob Public Health. 2016;11(7–8):1010–25. 26824463 10.1080/17441692.2015.1134614 PMC4983682

[9] Herbst JH, Jacobs ED, Finlayson TJ, McKleroy VS, Neumann MS, Crepaz N Estimating HIV prevalence and risk behaviors of transgender persons in the USA: a systematic review. AIDS Behav. 2008;12(1):1–17. 17694429 10.1007/s10461-007-9299-3

[10] Poteat T, Wirtz AL, Radix A, Borquez A, Silva-Santisteban A, Deutsch MB, et al HIV risk and preventive interventions in transgender women sex workers. Lancet. 2015;385(9964):274–86. 25059941 10.1016/S0140-6736(14)60833-3 PMC4320978

[11] Teh YK HIV-related needs for safety among male-to-female transsexuals (mak nyah) in Malaysia. Sahara-J. 2008;5(4):178–85. 19194599 10.1080/17290376.2008.9724917

[12] Farmer P Infections and inequalities: the modern plagues. Berkeley and Los Angeles: University of California Press; 2001.

[13] Farmer P On suffering and structural violence: a view from below. Race/Ethnicity: Multidiscip Global Perspect. 2009;3(1):11–28.

[14] Farmer P. An anthropology of structural violence 1. Curr Anthropol. 2004;45(3):305–25.

[15] Ahmed SM, Tomson G, Petzold M, Kabir ZN Socioeconomic status overrides age and gender in determining health-seeking behaviour in rural Bangladesh. Bull World Health Organ. 2005;83:109. 15744403 PMC2623805

[16] Beattie T, Shetty A, Devi Vanta U, Lowndes CM, Alary M, Bradley JE The evolution of female sex work In: Guntur A, eds. Pradesh: a qualitative study of HIV-related issues. Quebec, Canada: Centre Hospitalier affilié Universitaire de Quebec; 2009.

[17] Cusick L, Hickman M ‘Trapping’ in drug use and sex work careers. Drugs Educ Prev Policy. 2005;12(5):369–79.

[18] Ha R *Piloting a savings-led microfinance intervention with women engaging in sex work in Mongolia: A summary report*. Undarga 2014 [cited 2015 701]; Available from: http://blogs.cuit.columbia.edu/undarga/2014/07/08/piloting-a-savings-led-microfinance-intervention-with-women-engaging-in-sex-work-in-mongolia-a-summary-report/.

[19] Tsai LC, Witte SS, Aira T, Riedel M, Hwang HG, Ssewamala F “There is no other option; we have to feed our families… who else would do it?”: the financial lives of women engaging in sex work in Ulaanbaatar, Mongolia. Glob J Health Sci. 2013;5(5):41. 23985105 10.5539/gjhs.v5n5p41 PMC4041103

[20] Kennedy CE, Fonner VA, O’Reilly KR, Sweat MD A systematic review of income generation interventions, including microfinance and vocational skills training, for HIV prevention. Aids Care. 2014;26(6):659–73. 24107189 10.1080/09540121.2013.845287 PMC3943565

[21] Witte SS, Aira T, Tsai LC, Riedel M, Offringa R, Chang M, et al Efficacy of a savings-led microfinance intervention to reduce sexual risk for HIV among women engaged in sex work: a randomized clinical trial. Am J Public Health. 2015;105(3):e95–102. 10.2105/AJPH.2014.302291 PMC4330842 25602889

[22] Tsai LC, Witte SS, Aira T, Altantsetseg B, Riedel M. Piloting a savings-led microfinance intervention with women engaging in sex work in Mongolia: further innovation for HIV risk reduction. Open Womens Health J. 2011;5:26. 24900163 10.2174/1874291201105010026 PMC4041298

[23] Sherman SG, Srikrishnan AK, Rivett KA, Liu S-H, Solomon S, Celentano DD Acceptability of a microenterprise intervention among female sex workers in Chennai, India. AIDS Behav. 2010;14(3):649–57. 20352320 10.1007/s10461-010-9686-z

[24] Rotheram-Borus MJ, Lightfoot M, Kasirye R, Desmond K Vocational training with HIV prevention for Ugandan youth. AIDS Behav. 2012;16(5):1133–37. 21800180 10.1007/s10461-011-0007-y PMC3947885

[25] Pronyk PM, Hargreaves JR, Kim JC, Morison LA, Phetla G, Watts C, et al Effect of a structural intervention for the prevention of intimate-partner violence and HIV in rural South Africa: a cluster randomised trial. Lancet. 2006;368:1973. 17141704 10.1016/S0140-6736(06)69744-4

[26] Pronyk PM, Kim JC, Abramsky T, Phetla G, Hargreaves JR, Morison LA, et al A combined microfinance and training intervention can reduce HIV risk behaviour in young female participants. Aids. 2008;22(13):1659–65. 18670227 10.1097/QAD.0b013e328307a040

[27] Odek WO, Busza J, Morris CN, Cleland J, Ngugi EN, Ferguson AG Effects of micro-enterprise services on HIV risk behaviour among female sex workers in Kenya’s urban slums. AIDS Behav. 2009;13(3):449. 18998204 10.1007/s10461-008-9485-y

[28] Wong Y Islam, sexuality, and the marginal positioning of pengkids and their girlfriends in Malaysia. J Lesbian Stud. 2012;16(4):435–48. 22978284 10.1080/10894160.2012.681267

[29] Glaser BG, Strauss AL The discovery of grounded theory. Chicago IL: Aldine; 1968.

[30] NVIVO NVIVO 11. 2016 Unknown [cited 2017 303]; 1]. Available from: http://www.qsrinternational.com/products_nvivo.aspx.

[31] Patton MQ Qualitative research and evaluation methods. Newbury Park, California: Sage; 2002.

[32] Safika I, Levy JA, Johnson TP Sex work venue and condom use among female sex workers in Senggigi, Indonesia. Cult Health Sex. 2013;15(5):598–613. 23472595 10.1080/13691058.2013.776112 PMC3683348

[33] Hoffman L, Nguyen HTT, Kershaw TS, Niccolai LM Dangerous subtlety: relationship-related determinants of consistency of condom use among female sex workers and their regular, non-commercial partners in Hai Phong, Viet Nam. AIDS Behav. 2011;15(7):1372–80. 20924782 10.1007/s10461-010-9819-4

[34] Manopaiboon C, Prybylski D, Subhachaturas W, Tanpradech S, Suksripanich O, Siangphoe U, et al Unexpectedly high HIV prevalence among female sex workers in Bangkok, Thailand in a respondent-driven sampling survey. Int J STD AIDS. 2013;24(1):34–38. 23512512 10.1177/0956462412472300 PMC4935540

[35] Huang Y, Henderson GE, Pan S, Cohen MS HIV/AIDS risk among brothel-based female sex workers in China: assessing the terms, content, and knowledge of sex work. Sex Transm Dis. 2004;31(11):695–700. 15502679 10.1097/01.olq.0000143107.06988.ea

[36] Malaysia DOS *Population Distribution and Basic Demographic Characteristic Report 2010* 2011 [058 2011]; [cited 2016.

[37] Couture M-C, Sansothy N, Sapphon V, Phal S, Sichan K, Stein E, et al Young women engaged in sex work in Phnom Penh, Cambodia, have high incidence of HIV and sexually transmitted infections, and amphetamine-type stimulant use: new challenges to HIV prevention and risk. Sex Transm Dis. 2011;38(1):33–39. 21085056 10.1097/OLQ.0b013e3182000e47 PMC3729941

[38] Kab V, Evans J, Sansothy N, Stein E, Claude-Couture M, Maher L, et al Testing for amphetamine-type stimulant (ATS) use to ascertain validity of self-reported ATS use among young female sex workers in Cambodia. Addict Sci Clin Pract. 2012;7(1):11. 23186171 10.1186/1940-0640-7-11 PMC3507647

[39] Ho HT, Le GM, Dinh TT Female sex workers who use amphetamine-type stimulants (ATS) in three cities of Vietnam: use and sexual risks related to HIV/AIDS. Glob Public Health. 2013;8(5):552–69. 23679831 10.1080/17441692.2013.790459

[40] Hail-Jares K, Choi S, Duo L, Luo Z, Huang ZJ Occupational and demographic factors associated with drug use among female sex workers at the China–Myanmar border. Drug Alcohol Depend. 2016;161:42–49. 26897586 10.1016/j.drugalcdep.2016.01.026

[41] Creswell JW, Miller DL Determining validity in qualitative inquiry. Theory Pract. 2000;39(3):124.

[42] Creswell JW Research design: qualitative, quantitative, and mixed method approaches. California, USA: Sage Publications; 2003.

[43] Payne G, Williams M Generalization in qualitative research. Sociology. 2005;39(2):295–314.

[44] Blankenship KM, Friedman SR, Dworkin S, Mantell JE Structural interventions: concepts, challenges and opportunities for research. J Urban Health. 2006;83(1):59–72. 16736355 10.1007/s11524-005-9007-4 PMC1473169

[45] Ghose T, Swendeman DT, George SM The role of brothels in reducing HIV risk in Sonagachi, India. Qual Health Res. 2011;21(5):587–600. 21266706 10.1177/1049732310395328

[46] Evans C, Lambert H Implementing community interventions for HIV prevention: insights from project ethnography. Soc Sci Med. 2008;66(2):467. 17920740 10.1016/j.socscimed.2007.08.030

[47] Swendeman D, Basu I, Das S, Jana S, Rotheram-Borus MJ Empowering sex workers in India to reduce vulnerability to HIV and sexually transmitted diseases. Soc Sci Med. 2009;69:1157. 19716639 10.1016/j.socscimed.2009.07.035 PMC2824563

